# Class II Malocclusion in Adult Patients: What Are the Effects of the Intermaxillary Elastics with Clear Aligners? A Retrospective Single Center One-Group Longitudinal Study

**DOI:** 10.3390/jcm11247333

**Published:** 2022-12-09

**Authors:** Roberto Rongo, Simona Dianišková, Antonio Spiezia, Rosaria Bucci, Ambrosina Michelotti, Vincenzo D’Antò

**Affiliations:** 1 School of Orthodontics, Department of Neurosciences, Reproductive Sciences and Oral Sciences, University of Naples Federico II, Via Pansini, 5, 80131 Naples, Italy; 2 Department of Orthodontics, Medical Faculty, Slovak Medical University, Limbová 12, 83303 Bratislava, Slovakia

**Keywords:** clear aligners, Class II, adults, elastics

## Abstract

Aim: To evaluate the dental effects of the treatment with clear aligners and intermaxillary elastics in adult patients with Class II malocclusion. Material and methods: A sample of 20 Class II patients treated with Invisalign aligners (5 M and 15 F; mean age of 27.6 ± 6.3 years) was included in this single-center one-group longitudinal study. Dental cast and cephalometric records were analyzed before (T0) and after treatment (T1). Data were analyzed with a *t*-test for paired data (*p* < 0.05). Results: There was a significant reduction of the Overjet (OVJ= −1.4 ± 0.2; *p* ≤0.001) and a retroposition of upper incisors (U1-NPo = −1.3 ± 1.7; *p* < 0.001). Furthermore, distalization of upper molars with an improvement of molar class (U6-PT Vertical = −0.93 ± 0.97; *p* < 0.001; Molar Relation = −0.75 ± 0.45; *p* < 0.001) was observed. A good control of the lower and upper incisor inclination was present, highlighted by the non-significant changes in these values (L1-GoGn = −0.12 ± 5.4; *p* = 0.923; U1-AnsPns = −1.1 ± 8.1; *p* = 0.551). In the lower arch, an increase in the intermolar diameter (0.6 ± 1.0; *p* = 0.01) was present. Finally, there were no statistically significant changes in all the skeletal variables (ANPg = 0.005 ± 0.687; *p* = 0.974; SN/MP = −0.47 ± 1.9; *p* = 0.298). Conclusions: Treatment with Invisalign aligners shows a reduction of the Overjet, a retroposition of the upper incisors, good control of the lower incisors, and an improvement of the molar relationship.

## 1. Introduction

In recent years, the increase in the use of clear aligners has been favored by wide advertising of the product, and by the growing aesthetic needs of patients, especially among adults who prefer aesthetic treatments that do not affect social relationships and lifestyle [1,2,3].

Aligner Therapy (AT) consists of an aesthetic orthodontic treatment that includes customized, removable devices. Studies have shown that this treatment does not interfere with a patient’s oral hygiene and consequently does not affect periodontal health [4]. The use of a virtual set-up is an important tool for providing a very detailed treatment plan and evaluating the different therapeutic alternatives. In addition, patients show good collaboration with AT [5].

Today, with advances in AT biomechanics and with the improvement in the characteristics of the material [6], new therapeutic opportunities have been possible and have further increased the diffusion of this approach. AT may also include the use of auxiliaries such as elastics and other intra- and inter-maxillary accessories in order to treat complex cases. The improved biomechanics of AT allows for achieving difficult dental movements such as the distalization of the upper molars [7,8].

In orthodontics, many solutions have been proposed for the treatment of Class II malocclusions, among which: mandibular advancement, orthodontic camouflage, upper distalization with miniscrews for orthodontic anchorage, orthognathic surgery, and fixed multibrackets therapy and intermaxillary elastics. Although the benefits of Class II correction with fixed therapy are numerous, this approach might involve some risks for periodontal tissue health in the anterior teeth, like gingival recession, and often causes aesthetic impairment and concerns to the individual [9].

The use of intermaxillary elastics with fixed multibracket is the most used therapy to treat Class II dental malocclusion [10]. Effects of therapy with Class II elastics are mainly dentoalveolar: in the upper arch, extrusion and retrusion of the upper incisors with uncontrolled lingual inclination is observed, while, at the lower arch, buccal inclination and intrusion of lower incisors, and extrusion and mesialization of mandibular molars can be found. Furthermore, during the treatment, a temporary increase in occlusal plane angle can be observed, which seems to return to its original values at the end of the therapy [11].

Although the application of Class II elastics with multibracket fixed appliances is currently the most used treatment approach, the use of Class II elastics with aligners are extremely frequent in daily clinical practice. Nevertheless, still scarce information on the effects of this treatment in adult Class II patients is available [10]. A recent study performed in growing patients showed better control of lower incisor inclination using AT as compared to fixed multibracket therapy [12], but no information is present concerning adult treatment. Therefore, the purpose of this investigation was to assess the dentoalveolar effects of AT during the correction of Angle Class II division 1 malocclusions in a group of adult patients.

## 2. Materials and Methods

This was a retrospective single-center, one-group longitudinal study.

The study protocol complied fully with the principles of the Helsinki Declaration and was approved by the Ethics Committee of the University Federico II (352/21).

### 2.1. Sample

The study included data from 20 patients (mean age 27.9 ± 7.5 years, age range 20–43 years), of which 15 females and 5 males were treated between 2015 and 2020 at the School of Orthodontics of Bratislava Comenius (Slovakia). Data were analyzed at the School of Orthodontics, Department of Neuroscience, Reproductive Sciences and Oral Sciences of the University of Naples Federico II (Italy).

The inclusion criteria for the selection in the study were the following:

- Patients aged 18 or over at the beginning of the treatment.
- At least End-to-End Class II molar relationship.
- Permanent dentition.
- Overjet ≥ 4 mm measured on the most proclined tooth.
- Patients that finished the treatment with a good alignment and a Class I molar and canine relationship.

Subjects were excluded from the study if they had one of the following exclusion criteria:

- Craniofacial abnormalities.
- Congenital syndromes of the craniofacial area.
- Periodontal disease.
- Temporomandibular disorders.

### 2.2. Clinical Protocol

All patients underwent non-extractive orthodontic treatment.

Clear aligners (Invisalign^®^, Align Technology, Santa Clara, CA, USA) were used for this clinical treatment. Patients were instructed to wear full-time intermaxillary elastics 1/4” 6 oz from the precision cuts on the upper aligner connected to the tubes bonded on the first lower molars, starting from the third aligner.

Four sets of aligners were delivered to each patient, and they were instructed to change the aligner every 7 days. Regular check-ups were performed every 4 weeks. The average treatment time was 1.6 ± 0.6 years (treatment time range 1–2.8 years).

### 2.3. Measurements and Timing

**Figure 1:**
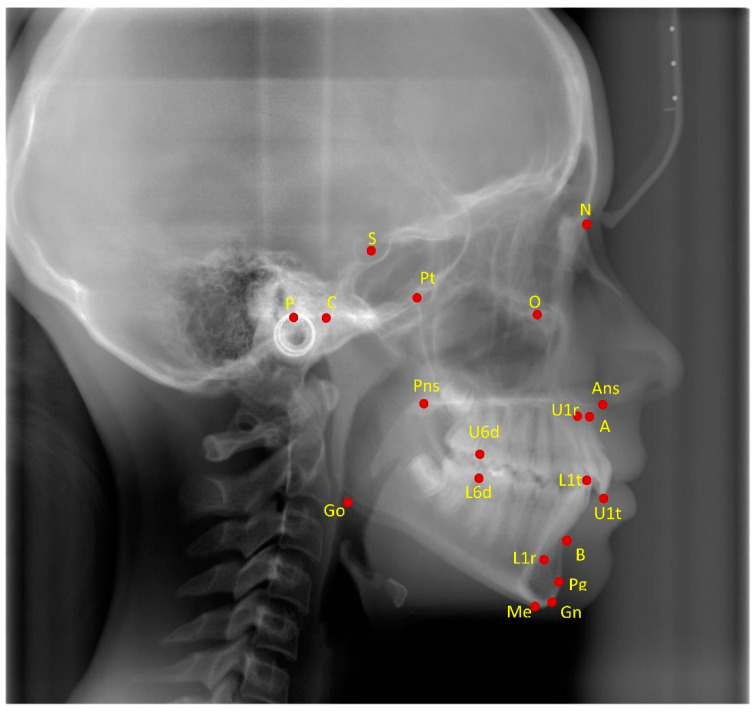
Cephalometric landmarks. N Nasion, S Sella, P Porion, Co Condylion, Pt Pterion, Or Orbitale, Pns Posterior nasal spine, Ans Anterior nasal spine, A Point A, B Point B, Pg Pogonion, Gn Gnathion, Me Menton, Go Gonion, U1r Root Apex Upper central incisor, U1t Tip Upper central incisor, L1r Root Apex Lower central incisor, L1t Tip Lower central incisor, U6d Distal Upper first molar, L6d Distal Lower first molar.

Measurement and timing were similar to those adopted in a previous study on growing patients (for detailed information, please see Dianiskova et al., 2021 [12]). The skeletal and dental changes resulting from the treatment were evaluated by means of cephalometric analysis on lateral cephalograms, taken in natural head position and in centric occlusion at the beginning (T0) and at the end of the treatment (T1) (Figure 1, Table 1). All lateral cephalograms were taken using the same radiographic appliance (ART Plus C; Ajat, Espoo Finland). Lateral cephalograms were randomly submitted to the examiner for cephalometric analysis, and the dates of the radiographic examination were concealed. Molar relationship, amount of lower crowding (from second bicuspids to second bicuspids), and amount of interproximal reduction (IPR) at the lower anterior teeth (from canine to canine) were assessed using digital dental casts at the beginning and at the end of treatment. The digital dental casts were analyzed by means of Autodesk MeshMixer (Autodesk INC. San Francisco, CA, USA). Crowding was defined as the difference between the sum of the mesiodistal widths of teeth from the second bicuspids to the second bicuspids at the lower jaw and the arch length. The mesiodistal widths of teeth from cuspid to cuspid at the end of treatment were also used to estimate the amount of IPR. In particular, for each tooth, the difference in mm between the mesiodistal width at T0 minus the mesiodistal width at T1 was calculated. The molar class relationship was studied by analyzing the digital cast in occlusion. Intermolar and intercanine distances were measured only at the lower arch, from the mesiobuccal cusp of the left first molar to the mesiobuccal cusp of the right first molar and from left to right cuspid.

### 2.4. Intra-Observer Reliability

By selecting 20 random cephalograms, intra-observer reliability was evaluated. The same examiner re-evaluated a series of measurements after 4 weeks. The intra-observer reliability for all measurements was calculated using Dahlberg’s formula and the intraclass correlation coefficient.

Systematic differences between duplicated measurements were tested using a paired Student’s *t*-test with the type I error set at 0.01.

### 2.5. Statistical Analysis

For each parameter collected, descriptive statistic (mean and standard deviation for continuous data) was reported.

The sample size calculation was based on the main outcome: lower incisor proclination (IMPA).

Considering alpha = 0.05, beta = 0.20, *t*-test for paired data, and an effect size of 0.7 (L1/GoGn mean difference = 4.3° grouped standard deviation = 5.8°) [12], at least 19 patients were required.

A *t*-test for paired data was used to compare each variable within the two-time points. Data distribution was analyzed by means of the Shapiro–Wilk test. A linear regression analysis was performed to evaluate the association between IPR, SNA_T0, Overjet_T0, Age, Sex, L1 to mandibular plane_T0, and L1 to mandibular plane_(T1-T0). Only values of *p* < 0.05 were considered significant. All statistical analyses were performed using the Standard statistical software package (SPSS version 22.0, IBM SPSS, Armonk, NY, USA).

## 3. Results

The intra-observer reliability ranged between 0.32° and 1.22° for cephalometric angular measurements and between 0.52 mm and 1.02 mm for linear measurements. There was no systematic error for any measurement (Student’s *t*-test: *p* > 0.01). ICC ranged between 0.770 to 0.999. At the beginning of the treatment, the group showed, on average, less than 2 mm of crowding (−1.94 ± 3.5).

Dentoalveolar changes were the main effects observed following AT (Table 2). Clinically and statistically significant overjet correction (OVJ = −1.4 ± 0.2; *p* < 0.001) was observed. Also, good control of the inclination of lower incisors, without any significant proclination (L1-GoGn = −0.12 ± 5.4; *p* = 0.923), was found. A significant retroposition of upper incisors compared to basal bone was present (U1-NPo = −1.3 ± 1.7; *p* = 0.001) with good control of the inclination (U1-AnsPns = −1.1 ± 8.1; *p* = 0.551). Furthermore, a statistically significant improvement of the molar class was found (Molar Relation = −0.75 ± 0.45; *p* < 0.001) associated with a significant distalization of upper molars (U6-PT Vertical = −0.93 ± 0.97; *p* < 0.001). The lower arch slightly expanded (intermolar diameter = 0.6 ± 1.0; *p* = 0.001), and the amount of IPR was 2.75 ± 1.57.

Regarding skeletal variables, no significant differences were observed in all the studied sagittal and vertical outcomes. In particular, no significant changes were observed at the end of treatment (ANPg = 0.005 ± 0.7; *p* = 0.974). Similarly, there was no statistically significant change in the lower jaw inclination (SN/MP = −0.47 ± 1.9; *p* = 0.298).

The linear regression analysis showed a statistically significant relation between L1 to mandibular plane_T0 and L1 to mandibular plane_(T1-T0), supporting a negative association (B = −0.83; CI 95%-1.35; −0.31; *p* = 0.004, Table 3).

## 4. Discussion

This study assesses the efficacy and the effects of AT for the resolution of mild Class II malocclusion in adult patients.

Given the limitations of the current study, AT represents a good treatment option for Class II malocclusion, thus reducing the OVJ, improving the molar relationship and retropositioning upper incisors, and having a good control on upper and lower incisors inclination. The control of the inclination of lower incisors is a key factor in this kind of treatment, considering that in dentoalveolar Class II corrections with inter-maxillary elastics the proclination of lower incisors is often undesired [11]. Indeed, the correction of Class II malocclusion with increased overjet and overbite may take advantage of the positive inclination of the lower incisors, but in some cases, such as dolichofacial subjects with thin, cortical bone of the mandibular symphysis, this movement can cause periodontal problems [9]. The results of the current study on incisor inclination are consistent with those reported in the previous study conducted on growing individuals [12] and thus support the use of AT in patients in which buccal inclination of lower incisors is an unwanted movement. However, it has to be underlined that in growing patients, the correction of the sagittal malocclusion is also related to the possibility of differential mandibular growth [13]. Therefore our hypothesis is that a limited amount of Class II relationship can be corrected without losing anchorage in the lower arch with the possibility of controlling the incisors’ position using the IPR.

The present study confirmed that Class II elastics and clear aligners are able to produce a slight distalization of upper molars. Several different approaches have been previously proposed for upper molar distalization, but all of them showed some critical issues, such as the loss of anchorage, the lack of patients’ compliance, and the development of some adverse effects on dentition [14].

The use of Class II intermaxillary elastics is often associated with a significant clockwise rotation of the occlusal plane, extrusion of the maxillary incisors, and a worsening of smile aesthetics due to the possible extrusion of the upper incisors associated with a greater gum exposure [11,15]. According to the results of the present investigation, clear aligners seem to provide adequate control of both extrusion and inclination of upper incisors, thus avoiding the increase of gum exposure. In addition, good control of the clockwise rotation of the mandible was also found. Indeed, another possible advantage of the AT is the so-called “bite effect”, which may offer greater control on the stability of the mandibular plane by preventing molar extrusion, which is a very common effect in the treatment with Class II elastics and fixed appliances. In particular, this was supported in the current sample by the absence of significant changes in the SN/MP values. These results are in accordance with previous studies performed with clear aligners, showing good predictability in the distalization of upper molars, with adequate control of the mandibular plane orientation and of the incisal extrusion [7,16,17]. Consequently, AT could be considered an efficient alternative for the distalization of upper posterior teeth without worsening upper incisors extrusion. The good control of teeth extrusion and inclination offered by AT might be associated with a homogeneous distribution of forces on the aligner shape as compared to the brackets and to the increased rigidity of the system that locks the arch shape and length. These factors might also be responsible for the good maintenance of the upper incisor inclination. Considering that the loss of upper incisors torque is one of the major problems of the treatments with Class II elastics and fixed multibracket appliances, due to the biomechanics of the elastics and the archwire/bracket play, aligners seem to offer an advantage whenever maintenance of upper incisors inclination is critical. In summary, it can be assumed that the structure of the AT avoids the proclination of the lower incisors and the retroclination of the upper incisors due to the Class II elastics, with an improvement of the molar relationship due to the distalization and the distal rotation of the upper molar.

Besides intermaxillary Class II elastics, other auxiliaries have been suggested in combination with fixed appliances to correct Class II malocclusions. The Forsus Fatigue Resistance Device (Forsus FRD^®^; 3MUnitek, Monrovia, CA, USA) is a non-compliant option for Class II Division 1 treatment. However, the observed proclination of lower incisors following treatment with these devices is similar to that provided with intermaxillary elastics [18]. Another non-compliant option is the Herbst^®^ appliance [19] which also presents a significant increase in lower incisors proclination (IMPA = 3.2° ± 12.8°) [20]. Another treatment option for severe Class II non-growing individuals is orthognathic surgery, aiming at reducing the profile convexity and improving the soft tissue appearance and the airway dimensions, which represent major impairments in adult patients [21,22].

The use of Class II elastics with aligners is widely adopted in clinical practice thanks to the good mechanical and chemical characteristics [23,24], the opportunity to monitor the dental movements during treatment [25], the preference for using removable appliances that require fewer emergency visits and allow better oral hygiene [26,27]. But although the use of aligners is very spread, according to the authors’ knowledge, small evidence in the literature on the effects of these devices with Class II elastics could be found. Patterson and co-authors compared the results of 40 Class I cases versus 40 Class II cases treated with Invisalign. The authors concluded that the treatment failed in the improvement of the Class II relationship and in the reduction of the overjet. The authors underlined that it was not possible to evaluate patients’ compliance with aligners or elastics and that the comparison was performed at the end of the first set of aligners [28]. Our study included only cases that finished in Class I molar and canine relationship at the end of the treatment. This choice was made as the aim of the investigation was to assess the possible effect of AT in achieving a good treatment result without considering possible confounding factors such as patient compliance or final occlusion. It would be important for future studies to deepen this topic to provide clear indications on the use of AT and Class II elastics. Our study could be considered as a good starting point, but with the limitation of being a retrospective study. In particular, it was not possible to evaluate patient compliance in wearing the elastics, but the final Class I occlusion confirmed that the patients were sufficiently compliant with their therapy, including both aligners and intermaxillary elastics. Moreover, some additional aspects could have influenced the final inclination of the incisors, such as the amount of crowding, the amount of IPR, and the correction of the Curve of Spee. However, in the current sample, it was found that both the initial degree of crowding and the amount of IPR had not influenced the final incisors’ proclination, while the Curve of Spee was not evaluated.

## 5. Conclusions

The present data suggest that treatment with aligners combined with Class II intermaxillary elastics may prevent undesired inclination of lower incisors (L1/GoGn°) and provide adequate control of upper incisor inclination during retropositioning, with no effects on sagittal and vertical skeletal cephalometric variables

Therefore, clear aligners might be a useful tool in the treatment of Class II malocclusion in adult patients when a limited amount of Class II relationship correction is needed without a significant proclination of the lower incisors. However, considering that this is a retrospective single-center, one-group longitudinal study, further studies are needed to support these findings.

## Figures and Tables

**Table 1:** Analyzed cephalometric variables.

Sagittal Skeletal	Interdental
SNA (°)	Overjet (mm)
SNPg (°)	Overbite (mm)
ANPg (°)	Molar relation (mm)
WITS (mm)	**Maxillary dentoalveolar**
Co-Gn (mm)	U1 to palatal plane (°)
**Vertical skeletal**	U1-NPo (mm)
SN to palatal plane (°)	U6-PT Vertical (mm)
SN to mandibular plane (°)	**Mandibular dentoalveolar**
Palatal plane to mandibular plane (°)	L1 to mandibular plane (°)
CoGoMe (°)	
Co-Go (mm)	

**Table 2:** Descriptive statistics and statistical analysis T1 vs. T0. The significance level was set at *p* < 0.05. Data are reported as mean ± standard deviation (SD) and 95% confidence interval (95% CI).

	Aligners T0 N = 20			Aligners T1 N = 20			*t* Test	T1 vs. T0	
Cephalometric Measures	Mean	SD	95% CI	Mean	SD	95% CI	*p*	Difference	SD
**Sagittal Skeletal**									
SNA (°)	81.4	2.9	80.1; 82.8	81.6	3.3	80.1; 83.1	0.581	0.14	1.1
SNPg (°)	78.2	4.3	76.3; 80.1	78.4	4.8	76.2; 80.6	0.454	0.20	1.2
ANPg (°)	3.2	2.6	2.1; 4.4	3.3	2.7	2.0; 4.5	0.974	0.005	0.7
Wits (mm)	1.9	2.7	0.7; 3.2	1.3	1.7	0.4; 2.1	0.348	−0.68	3.1
Co-Gn (mm)	108.1	9.5	103.8; 112.5	107.8	7.8	104.2; 111.4	0.766	−0.28	4.2
**Vertical Skeletal**									
SN/PP (°)	8.3	2.6	7.1; 9.5	8.1	3.2	6.6; 9.3	0.602	−0.23	2
SN/MP (°)	30	7.9	26.4; 33.7	29.6	8.7	25.5; 33.5	0.298	−0.47	1.9
PP/MP (°)	21.7	7.7	18.1; 25.2	21.3	7.6	17.8; 24.8	0.457	−0.35	2
CoGoMe (°)	123	8.9	119; 127.1	121.6	8.3	117.8; 125.4	0.251	−1.42	5.3
Co-Go (mm)	60	8.8	55.9; 64	60.1	8	53.5; 63.8	0.866	0.18	4.8
**Interdental**									
Overjet (mm)	4.6	0.8	4.3; 5	3.2	0.7	2.9; 3.5	0.001	−1.4	0.6
Overbite (mm)	3	1.7	2.2; 3.9	2.6	0.7	2.3; 3	0.234	−0.4	1.4
Molar Relation (mm)	0.5	0.7	0.1; 0.8	−0.27	0.5	−0.5; −0.02	0.001	−0.75	0.4
**Maxillary dentoalveolar**									
U1/PP (°)	109	9.2	104.9; 113.3	108	5.7	105.4; 110.6	0.551	−1.1	8.1
U1-NPo (mm)	8.2	4.2	6.2; 10.2	6.9	3.4	5.3; 8.4	0.001	−1.3	1.7
U6-PT Vertical (mm)	18	3.6	16.3; 19.6	17	4.1	15.1; 18.9	0.001	−0.93	0.9
**Mandibular dentoalveolar**									
L1/GoGn (°)	101.2	5.9	98.4; 103.9	100	6.1	95.8; 100.6	0.548	−1.30	8
Intercanine diameter (mm)	25.6	1.7	24.8; 26.4	26.3	2.7	25.1; 27.6	0.176	0.70	2.2
Intermolar diameter (mm)	47.8	3.1	46.4; 49.3	48.5	2.9	47.1; 49.8	0.001	0.60	1
Crowding	−1.9 ± 3.5								
Interproximal reduction	2.7 ± 1.6								

**Table 3:** Linear regression analysis between amount of IPR, SNA_T0, Overjet_T0, Age, Sex, L1 to mandibular plane_T0 and L1 to mandibular plane_(T1–T0). The significance level was set at *p* < 0.05. Beta coefficients (B) and 95% confidence interval (95% CI) are reported.

Cephalometric Measures	B	95% CI	*p*
IPR	−1.88	−4.33; 0.57	0.124
SNA_T0	0.80	−0.46; 2.06	0.198
Overjet_T0	0.51	−3.71; 4.72	0.802
L1 to mandibular plane_T0	−0.83	−1.35; −0.31	0.004
Age	−0.20	−0.73; 0.33	0.441
Sex	6.94	−0.92; 14.80	0.080

## Data Availability

The data presented in this study are available on request from the corresponding author.
